# The use of content and timing to predict turn transitions

**DOI:** 10.3389/fpsyg.2015.00751

**Published:** 2015-06-11

**Authors:** Simon Garrod, Martin J. Pickering

**Affiliations:** ^1^Institute of Neuroscience and Psychology, University of GlasgowGlasgow, UK; ^2^Department of Psychology, University of EdinburghEdinburgh, UK

**Keywords:** dialog, turn-taking, prediction, timing, content

## Abstract

For addressees to respond in a timely fashion, they cannot simply process the speaker's utterance as it occurs and wait till it finishes. Instead, they predict both when the speaker will conclude and what linguistic forms will be used. While doing this, they must also prepare their own response. To explain this, we draw on the account proposed by Pickering and Garrod (2013a), in which addressees covertly imitate the speaker's utterance and use this to determine the intention that underlies their upcoming utterance. They use this intention to predict when and how the utterance will end, and also to drive their own production mechanisms for preparing their response. Following Arnal and Giraud (2012), we distinguish between mechanisms that predict timing and content. In particular, we propose that the timing mechanism relies on entrainment of low-frequency oscillations between speech envelope and brain. This constrains the context that feeds into the determination of the speaker's intention and hence the timing and form of the upcoming utterance. This approach typically leads to well-timed contributions, but also provides a mechanism for resolving conflicts, for example when there is unintended speaker overlap.

## Introduction

How is it possible for most conversations to be so fluent and efficient? Interlocutors tend to respond coherently and appropriately to each other. But in addition, they do so in good time—they do not leave long gaps between contributions, nor do they speak simultaneously for more than a brief moment (Sacks et al., 1974). To understand this remarkable and almost universal ability for turn transitions, we need to explain the cognitive processes that take place in people's minds. So far, psychologists have developed detailed accounts of the moment-by-moment processes that underlie producing and comprehending in isolation, but have much less to say about the moment-by-moment processes involved in conversation. In this paper, we propose an account of those processes that specifically explains turn transitions.

## The nature of turn transitions

We begin with an example from Schegloff (1996, p. 73). Two students are talking on the phone about a book purchase, with [indicating overlapping speech, and numbers indicating noticeable pauses in seconds)^1^,^2^.

1.

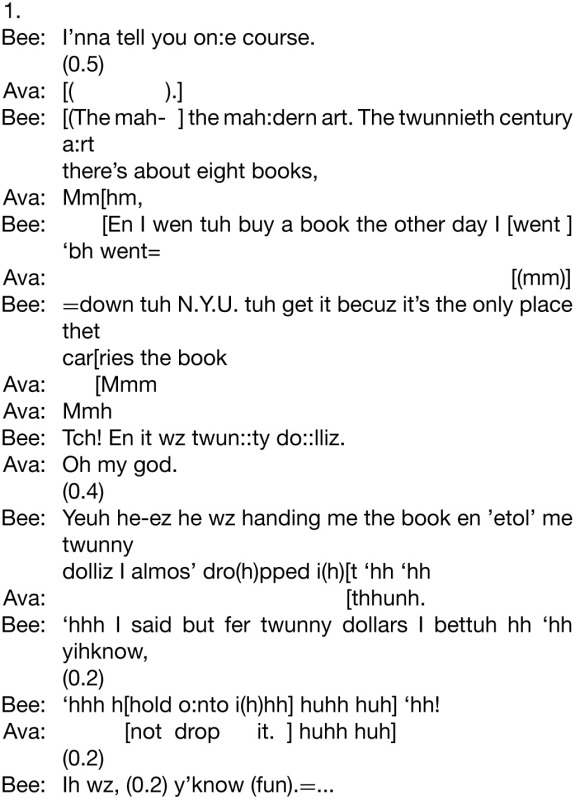



It is quite clear that the interlocutors contribute sequentially. On the one hand, any pauses are very short, but on the other, there is little overlap. In most cases, the overlap is not likely to interfere with comprehension, because people are able to speak and comprehend “backchannel” contributions such as *mm* (or listen and produce such contributions) at the same time. Somehow, the addressee must be able to know when to speak and when to be quiet, even though she does not know exactly what her partner is going to say.

Conversation analysts have very carefully analyzed what people do during conversations such as these (i.e., rather than highly ritualized or formulaic interchanges). Among other observations, Sacks et al. (1974, pp. 700–701) noted:

- Overwhelmingly, one party talks at a time.
- Occurrences of more than one speaker at a time are common, but brief.
- Transitions (from one turn to the next) with no gap and no overlap are common. Together with transitions characterized by slight gap or slight overlap, they make up the vast majority of transitions.
- Turn size is not fixed, but varies.
- What parties say is not specified in advance.
- Turn-allocation techniques are obviously used. A current speaker may select a next speaker (as when he addresses a question to another party); or parties may self-select in starting to talk.
- Repair mechanisms exist for dealing with turn-taking errors and violations; e.g., if two parties find themselves talking at the same time, one of them will stop prematurely, thus, repairing the trouble.

All of these observations are clearly relevant for the above example. Our goal is to explain such observations in cognitive-psychological terms. Our focus is on (c), and to some extent (b), (f), and (g). One important reason for (a) is presumably basic limitations on processing resources (it is very hard to produce and comprehend different messages at the same time). Observations (d) and (e) occur because conversations are generally unplanned and because people's goals vary (they may want to make small or large contributions) and may be affected by the conversation itself.

Sacks et al.'s (1974) work is based on English. Stivers et al. (2009) compared turn transitions for questions and responses across speakers of 10 diverse languages and found slight variation in distribution. But in all cases the most frequent interval was between 0 and 200 ms. In other words, conversationalists show a strong disposition to avoid overlap and to minimize silence between turns. They concluded that these properties of conversation constitute robust human universals (though cultural and linguistic factors lead to minor variations). So how is it possible for interlocutors to contribute with such short intervals between turns, while avoiding extensive overlap? How can the addressee prepare and execute an appropriate response while comprehending the speaker?

## The processes underlying turn transition

Given such intervals, addressees cannot simply wait for the speaker to end before preparing their response. First, it would of course take some time to determine that the speaker has ended. Second, many studies have demonstrated that producing a single word requires about 175 ms to access meaning, 75 ms for syntax, 205 ms for phonology, and 145 ms for phonetic encoding and articulation (Indefrey and Levelt, 2004; see Sahin et al., 2009, for somewhat different estimates). Even if these timings might be slightly different in conversation (rather than, for example, picture naming), it is clear that, in general, addressees must be able to estimate when the speaker's turn will end and begin response preparation several hundred milliseconds before that point.

This suggests that comprehension and production processes must be tightly interwoven. In fact, this assumption is quite controversial within the psychology of language, which tends to have studied comprehension and production in isolation and assumes that they involve largely independent mechanisms (Pickering and Garrod, 2013a). According to traditional accounts, dialog therefore can be characterized as serial monolog, in which the speaker produces and the addressee listens, and at the turn-transition point (i.e., transition relevance place) they switch roles and processes.

In fact, the serial monolog account suggests that speakers cannot prepare their utterances until they realize that their partner has completed (which may be later than the actual completion point). This would obviously be incompatible with Sacks et al. (1974) and Stivers et al. (2009). To avoid these problems, comprehenders would have to use ancillary mechanisms based on their comprehension systems to predict turn completions. These mechanisms would not be relevant for production, so they would have to begin preparing a response using their production systems in parallel to comprehension-based prediction. Moreover, they would have to determine the meaning of the complete utterance and then use this as a basis for generating an appropriate response.

These problems are, however, avoided if comprehenders use their production systems to make predictions and prepare their responses together. The mechanisms that they use to predict a speaker's final word, for example, are closely related to the mechanisms they themselves use to produce their response—or indeed to complete their partner's utterance if necessary (e.g., to help with word finding difficulty; A: *That tree has, uh, uh …* B: *tentworms*; Clark and Wilkes-Gibbs, 1986, p. 6). We now (1) specify the problem faced by the addressee; (2) discuss how addressees use predictions of timing and content to predict when the speaker will complete; and (3) discuss how the addressee can produce an appropriate and timely response.

## Managing fluent turn-transition requires predicting both speech content and timing

The addressee has to predict when the speaker is going to finish, and prepare an appropriate response. It would not be sufficient to prepare a response without predicting the end-point, because studies have shown that producing a prepared linguistic response to a cue takes several hundred milliseconds. For example, Ferreira (1991) had participants memorize and then produce sentences following a cue, and found response times of 500 ms or more. It takes at least as long to initiate prepared picture naming (e.g., Piai et al., 2011). Similarly, simply predicting the end-point would merely remove any time needed to determine that the speaker had ended, but not help with response preparation.

In fact, De Ruiter et al. (2006) showed that listeners could accurately estimate when a speaker's conversational turn was about to end. Their participants heard turns taken from recordings of natural conversations and indicated precisely when they thought the turn would end. The average response was about 186 ms before the turn actually ended. Interestingly, turn-ending estimates were not affected by flattening the pitch contour of the speech, but were dramatically affected when the lexico/semantic content was removed. This suggests that listeners used the content to predict turn endings. It is of course possible that other sources of prosodic information might affect estimates; for example, future investigations could test whether addressees are sensitive to rising intonation when responding to a question. In a subsequent study, Magyari and De Ruiter (2012) had another group of listeners predict the remaining words in De Ruiter et al.'s turn fragments. They found turn-end judgments were more precise when those listeners made accurate predictions than when they did not. An obvious explanation of these findings is that people's predictions of words constitute a factor (alongside speech rate) that is used to predict turn endings.

Experimental studies have shown that people predict aspects of upcoming words such as their syntactic features (e.g., Van Berkum et al., 2005) and their sound (e.g., DeLong et al., 2005), and that they also predict upcoming constituent structure (Staub and Clifton, 2006). Indeed, many theoretical accounts assume that comprehension is an inherently predictive process (Hale, 2001; Levy, 2008). We therefore propose that people can draw on local predictions of words and other linguistic information to predict turn endings.

It may also be possible to make predictions relating to semantics and pragmatics over a much longer period. The semantics of the context will place great constraints on the upcoming content (e.g., whether the speaker is likely to talk about food, work, holiday plans, or whatever). Of course, such information can come from the current utterance (e.g., *Changing the subject, I'm hungry—what would you …*). Sometimes this information will only be apparent just before the prediction is needed, but often the relevant words occur early in the utterance, or in a previous utterance. In other cases, the information comes from the non-linguistic context (e.g., an unfolding event such as a parade), or from shared background knowledge (i.e., common ground). Usually, this information is available well before the prediction is needed. The addressee also benefits from determining the speaker's speech act before it is complete, because whether the speaker is producing a statement, question, or command may help determine the upcoming length of the utterance. (As we discuss later, determining the speech act is also critical to preparing a response).

From these sources of information, the addressee could predict what the speaker is likely to say. These predictions could include determining how much the speaker has left to say, as well as what the speaker is talking about. But to determine when the speaker will finish, the addressee has to combine these predictions with information about the speaker's speech rate. As our focus is on turn-transition, we now consider prediction of remaining content (what the speaker has left to say) and precise timing (when the speaker will finish). We then show how these predictions feed into the content and timing of the response itself.

## Using prediction-by-simulation in turn transition

Now we account for addressees' ability to predict turn-ending and deliver an appropriate and timely response. To do this, we draw on the integrated account of production and comprehension developed by Pickering and Garrod (2013a). This account is broadly compatible with other integrated accounts, which typically relate to language learning or distribution as well as language processing, such as the *P-Chain* framework (Dell and Chang, 2014) and the *Production-Distribution-Comprehension* account (MacDonald, 2013), as well as by evidence that prediction during comprehension engages production processes (Federmeier, 2007).

To predict the speaker's utterance, we proposed that the addressee attempts to determine the speaker's intention and uses that intention to predict what the speaker would say. For our purposes, the two aspects of this account that we need to consider are (1) that the addressee combines interpretation of the context and covert imitation of the speaker's prior utterance to estimate the intention; and (2) that the addressee uses the intention to predict the speaker's completion in the same way that the addressee would predict his or her own utterance if speaking at that point (though adjusting for differences between the speaker and the addressee). This process is known as *prediction-by-simulation* and works because the comprehender has similar representations and mechanisms to the producer. (Comprehenders may also use *prediction-by-association*, which relies on past experiences during comprehension; see Pickering and Garrod, 2013b, for discussion).

Consider a situation in which a mother is cooking dinner and her son comes into the kitchen and turns to speak. Based on the context (the food, the time, knowledge of her son's habits) but without any utterance, she estimates that his intention is to ask what is for dinner. But he then says *What are we going to do after …* and she now combines the context and the utterance to derive an (updated) intention—that he is producing a question in which the only missing element is something referring to dinner. Pickering and Garrod (2013a) assume that she represents his intention and that this constitutes her own “production command,” which sets off the processes that she would use to complete the utterance herself (adjusting for differences between herself and her son). This means that she converts the prior utterance into a production representation via “covert imitation,” which is then compatible with the format of the intention.

To understand how addressees predict speakers' utterances, we first note that Pickering and Garrod (2013a) argued that speakers predict their own utterances, using so-called *forward models*. For example, it may take several hundred milliseconds to start naming an object (e.g., Piai et al., 2011), but well before this, speakers can construct representations of what they believe they will say and what they will experience themselves saying. Psycholinguistic evidence for this claim comes from the finding that speakers are affected by the contextual probability of a target word or phrase given the preceding context. If the probability is higher, the speakers are more likely to produce a reduced form (Aylett and Turk, 2004) or to omit an optional word such as the complementizer *that* (Jaeger, 2010). This suggests that the speaker is sensitive to the probability of the target given the context, before uttering the target, and therefore has predicted the target by this point.

Pickering and Garrod (2013a) based their account on the mechanisms of action control, in which people predict movements before they occur and while they are occurring (and use their predictions to make corrections on-line; e.g., Wolpert, 1997). It assumes that people learn the relationship between their intentions and the outcomes (e.g., speech or arm movement), so that the forward model can be computed independently of the implementation of the action. It also assumes that people represent the inverse model of this relationship between outcomes and intentions on the basis of the forward model. They can then use the paired forward-inverse models to predict the outcomes of their actions (via forward models) and subsequently modify those actions when necessary (via inverse models), with both the learning and the on-line control being driven by prediction error minimization. Theories of speech production make such claims about syllables and phonemes (Hickok et al., 2011; Tourville and Guenther, 2011). Pickering and Garrod (2013a) make the more general claim that speakers can concurrently predict at the full range of linguistic levels, such as semantics, syntax, and phonology, and that they also make predictions about timing.

Following this, Pickering and Garrod (2013a) argued that comprehenders predict other people's utterances, again using forward-inverse model pairings. For example, if they believe that their partner is about to name an object, they can construct representations of what they believe their partner will say and what they will experience their partner saying. This is compatible with theories of action perception, in which people predict their partner's unfolding movements (Wolpert et al., 2003; Oztop et al., 2005). To do this, Pickering and Garrod argued that comprehenders covertly imitate the speaker, derive the (putative) intention of the speaker (using a combination of context and inverse model), use that intention to derive their upcoming intention, and treat this upcoming intention as the input to the forward models that predict the upcoming utterance, again at different linguistic levels (see also Pickering and Garrod, 2014). This proposal means that predicting another person's utterance involves the same predictive mechanism used to predict one's own utterance.

Pickering and Garrod (2013a) explained dialog as a form of joint action in which both interlocutors predict both their own and their partner's utterances. The addressee can predict the speaker's unfolding utterance and how he might respond to that utterance. The speaker similarly can predict how she will continue and how her partner might respond. Well-aligned interlocutors (Pickering and Garrod, 2004) tend to make the same predictions as each other. Moreover, Pickering and Garrod (2014) proposed that interlocutors monitor the quality of these predictions and use the discrepancies between predicted and actual utterances (by themselves and their partners) to control the flow of the dialog.

We propose that interlocutors make two different types of prediction during comprehension, relating to content and timing. The basis for content prediction is the processes of language comprehension typically investigated in psycholinguistics, and involves the extraction of phonology, syntax, and particularly semantics that can be derived from the speaker's utterance. From these representations, the comprehender can predict the phonology, syntax, and semantics of the upcoming utterance. The basis for timing prediction is the speaker's speech rate, which the comprehender can use to predict the rate of the upcoming utterance. We propose that these mechanisms are distinct, but that they can influence each other and be combined for various purposes. We now demonstrate how they can be used to predict turn-endings. At the end of the paper, we illustrate how they can be combined for other purposes, for example to resolve ambiguities (e.g., Dilley and Pitt, 2010).

To return to our example, the mother uses context to determine the boy's putative intention before he starts to speak and predicts that he will produce a fairly short question asking about what is for dinner. After the boy begins to speak, she revises her prediction by combining context with her covert imitation of the boy's incomplete utterance *What are we going to do after …* (a process that is in fact informed by her monitoring the discrepancy between his incoming utterance and her prior prediction). She therefore covertly imitates the boy's utterance, derives the boy's intention in producing *What are we going to do after* and derives her belief about his upcoming intention, which we assume is to produce the word *dinner* and then stop. She then predicts aspects of the form of *dinner* (e.g., main meal, noun, /dinər/, rising intonation, two syllables).

Note that Pickering and Garrod (2013a) argued that forward models are likely to be impoverished—not containing all of the information included in the implemented representations underlying actual speech (see several commentaries and Pickering and Garrod, 2013b, for discussion). By repeatedly producing utterances as a result of intentions, the speaker learns different intention-utterance regularities. She can draw on different regularities depending on the situation—for example, predicting the semantic class of the upcoming word (e.g., when predicting whether a speaker is going to suggest one of a set of restaurants) or the initial sound (when predicting whether the speaker is going to suggest a particular restaurant, e.g., *Kalpna*). The speaker predicts different aspects of the upcoming utterance on different occasions. Such flexibility clearly makes the forward models more useful for aiding fluency, but it also means that we cannot determine which aspects of an utterance will be represented on a particular occasion. In Alario and Hamamé's (2013) terms, we assume that the “opt-out” is circumstantial rather than systematic. For example, predictions may contain “fine-grained phonetic detail,” contra Trude (2013); see Pickering and Garrod (2013b, p. 379).

Quite separately, she determines his speech rate, which we assume is in terms of syllables, say 170 ms/syllable. Below, we discuss evidence both that speakers compute speech rate in terms of syllables and that they entrain on syllable rate. The boy's mother therefore assumes (without further computation) that the upcoming speech rate will also be 170 ms/syllable. Let us assume that her “target” is to leave a one-syllable gap between her son's contribution and her own (corresponding to what Schegloff, 2000, calls a *beat*). To determine point of initiation, she therefore estimates the length in syllables of her son's predicted completion (2) plus the gap (1), and multiplies them by syllable time (i.e., 3 × 170 ms = 510 ms). At the same time, she constructs linguistic representations for *What are we going to do after dinner* (i.e., including *dinner*), and uses them to prepare an appropriate response (e.g., *Play football*, which is syntactically and semantically appropriate). This preparation involves extension of the forward model to incorporate self- as well as other-prediction, and also involves the implementer—in other words, actual accessing of linguistic representations such as the lexical entries for *play* and *football*. This allows her to utter *Play football* after a one-syllable interval, assuming that he does utter *dinner* and takes 340 ms to do so.

Comprehenders might predict their partner's penultimate word and final word (both in terms of timing and content). Making these two predictions at the same time does not lead to resource competition because they are two compatible predictions, as they follow from the same process of covert imitation: one is the result of production command that would be used to predict the next word [*i*_*B*_ (*t* + 1) in the terms of Pickering and Garrod, 2013a], and the other the result of production command that would be used to produce the word after that (*i*_*B*_ (*t* + 2)). For example, they might predict a completion of *after* (in 340 ms) and *dinner* (in 640 ms). They do not compete for resources. We have also noted that comprehenders make predictions about their partner's completion and their own response (though of course they need to “tag” whether a specific prediction is about themselves or their partner). For example, they might predict their partner's final word *dinner* in 340 ms and their own response *Play football* in 510 ms. If these predictions are compatible, they will also not compete for resources. This will be true if the comprehender is well-aligned with the speaker, something that is likely to be the case in a simple question-answer case such as this. Of course, if someone is trying to comprehend a speaker while preparing an unrelated utterance (e.g., at a “cocktail party”), the self- and other-predictions are unlikely to be aligned and processing difficulties may ensue.

Note also that comprehenders may use forward models to predict multiple alternatives, weighted according to their likelihood (e.g., Wolpert and Kawato, 1998). Such multiple predictions are particularly valuable during comprehension, because the speaker may often produce one of many alternatives (e.g., *dinner, supper, the meal*). In fact, there is some evidence for parallel prediction in both ERP studies (DeLong et al., 2005) and corpus-based investigations of reading time (Smith and Levy, 2013). Such parallel prediction does not appear to be resource-intensive (as it is in many dual tasks).

Importantly, the content and timing predictions are combined, but they remain separate predictions. The comprehender does not construct a single (indivisible) representation of timing and content. This means that the comprehender can change either timing or content as necessary. For example, the boy might not stop after *dinner* but produce further words, or perhaps speak slowly or disfluently. If so, the mother would need to alter prediction of timing but not content. Alternatively, the boy might (unexpectedly) say *swimming* rather than *dinner*, in which case the mother would have to revise her interpretation (based on monitoring; Pickering and Garrod, 2014) but not timing. Below we explain how the flexibility induced by separate representations appears to be used in practice.

In more general terms, then, we assume that the addressee is constantly covertly imitating the speaker, and uses the process of covert imitation to make predictions about both the timing and the content of the speaker's utterance. This process supports alignment (Pickering and Garrod, 2004), so that the addressee's linguistic representations become more similar to those of the speaker, as well as entrainment of timing (see below). Sometimes the addressee predicts that the speaker is about to finish and that it would be appropriate for the addressee to take the floor. Alongside this, the addressee uses forward modeling to predict the speaker's concluding utterance and the addressee's own response (in a way that is aided by the alignment that has taken place). After the speaker finishes, and assuming that the addressee's prediction is correct or sufficiently close, the addressee speaks appropriately and at the appropriate time. We now discuss how entrainment of timing can take place, before turning to the question of how the addressee monitors the speaker's utterance and how difficulties can be managed.

## How does the addressee entrain timing with the speaker?

Arnal and Giraud (2012) argued that the brain implements predictions about timing and content in different ways. More specifically, predictions about the timing of sensory events are based on cortical oscillations in the low frequency range (*delta* band, 1–3 Hz; *theta* band, 4–8 Hz), whereas predictions about sensory content are based on higher frequency cortical oscillations (*gamma* band, about 30–60 Hz). Both auditory and pre-motor cortex reveal ambient neural oscillations in the *theta* range (Giraud et al., 2007). Those in the auditory cortex become entrained to theta oscillations in the speech envelope (see Gross et al., 2013; Zion Golumbic et al., 2013). These theta oscillations correspond to the frequency of the speaker opening and closing her mouth and hence the rate of her syllabic articulation (Chandrasekaran et al., 2009). According to Arnal and Giraud, predictive timing arises from this low-level mechanism of neural entrainment. In the presence of a fast speaker, the auditory cortex first adapts by increasing the rate of oscillations. These entrained oscillations then become predictive by creating periodical temporal windows for higher-order regions to read out encoded information (see also Kotz and Schwartze, 2010; Giraud and Poeppel, 2012). In other words, low frequency cortical oscillations come to predict the precise timing of critical speech events (at the level of the beginning and end of syllables).

There is now considerable empirical support for this with respect to speech perception. For example, Zion Golumbic et al. (2013) recorded ECoG (Electrocorticographic) activity in the auditory cortex as listeners attended to one of two speakers in a simulated “cocktail party” situation. They found that both the phase of low frequency cortical activity (i.e., delta and theta band) and the power of higher frequency cortical activity (high gamma) tracked the low frequency aspects of the speech envelope (i.e., the speech wave), for the attended but not the unattended speech. Follow-up analyses indicated that the higher frequency effects reflected evoked responses, whereas the low frequency effects reflected processes more closely related to perception. This latter finding suggests that low frequency speech tracking serves to limit the transfer of sensory responses to higher-order brain regions. As the low frequency phase of the attended and unattended speech is likely to be different, the listener can use phase tracking for selective attention.

Furthermore, they found that the precision of low frequency tracking increased steadily from the beginning to end of each attended utterance, consistent with a predictive process. Using a somewhat different approach, Gross et al. (2013) compared oscillatory MEG (Magnoencephalographic) signals in the cortex with those in the speech envelope for a 7-min narrative played both forwards and backwards. Mutual Information analyses revealed that low frequency (i.e., delta, theta band) cortical oscillations (in the right hemisphere auditory cortex) encoded the phase of low frequency oscillations in the speech envelope, whereas higher frequency (i.e., gamma) cortical oscillations (in the left hemisphere auditory cortex) encoded the energy of higher frequency oscillations in the speech envelope. Notably, the degree of oscillatory entrainment was much greater for the forward as opposed to the backward speech. Further, analyses of the forward speech established that transients (i.e., high energy bursts of sound) at the beginning of utterances reset the phase of low frequency cortical oscillations to bring it into line with the phase of low frequency oscillations in the speech envelope. Such resetting of the transients did not occur to the same extent for backward speech. This suggests that these effects reflected top-down predictive processing as opposed to bottom-up evoked responses.

These and related findings (see Ding and Simon, 2014) clearly implicate a low frequency oscillatory tracking system which represents current speech rate and predicts how it will unfold in the immediate future. Although the functional explanation for this entrainment process has been primarily related to syllabic parsing (Ghitza, 2011, 2013) or selective attention (Ding and Simon, 2012; Zion Golumbic et al., 2013), we propose that it may also play an important role in predicting when an interlocutor's turn will end and timing the addressee's response onset.

We hypothesize that cortical theta oscillations entrained during speech comprehension also influence the rate of speech production, probably mediated by mid-brain circuitry (see Giraud et al., 2007; Kotz and Schwartze, 2010). In other words, theta oscillations in auditory cortex entrain theta oscillations in premotor cortex, which in turn influence both the timing of the speech onset and rate of articulation. We assume that the rate and phase of oscillation play a causal role in such entrainment (though it is conceivable that entrainment results from some underlying pattern of neural activity that is highly correlated with oscillation). This is pertinent because (as we have noted), turn-transition involves more than detecting when an interlocutor's turn will end; it also involves initiating one's own turn in a timely fashion, with such inter-turn intervals reflecting the current speech rate. The finding that, during dialog, interlocutors' speech rates and turn transition times become entrained (Street, 1984) is consistent with coupling between the current speaker's rate and the subsequent speech rate of their partner (cf. Jungers and Hupp, 2009, for priming of speech rate in monolog). It is also consistent with Wilson and Wilson's (2005) proposal that the timing of turn-transitions is based on an underlying entrainment of syllabic speech-rate oscillations. Our proposal, therefore, is that interlocutors entrain theta oscillations in auditory cortex and premotor cortex, and that such entrainment underlies the coordination of comprehension and production in turn-taking.

**Figure 1:**
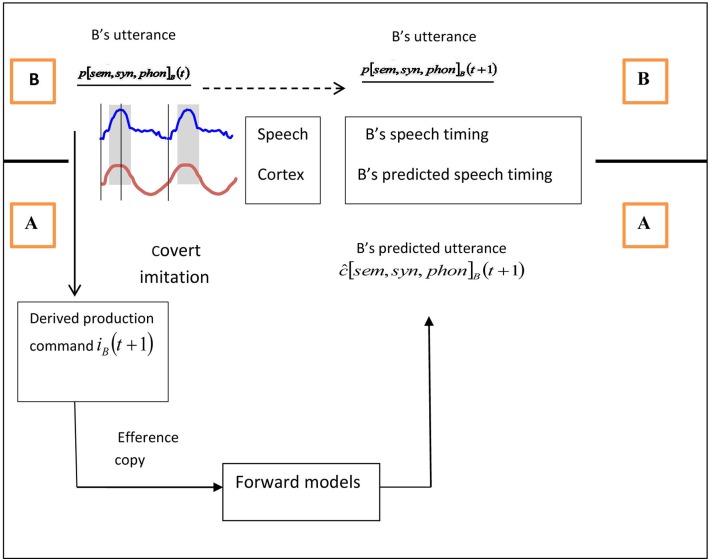
**A schematic illustration of the turn ending prediction mechanism, with *A* as addressee (below black line) and *B* as current speaker (above black line)**. Above the line, *B's* unfolding utterance content is shown as *p*[*sem*, *syn*, *phon*]_*B*_ (*t*) and *p*[*sem*, *syn*, *phon*]_*B*_ (*t* + 1), which refer to semantic, syntactic, and phonological representations of the current utterance (at time *t*) and the upcoming utterance (at time *t* + 1, with the underlining indicating that they are *B*'s representations; see Pickering and Garrod, 2013a). The timing of *B's* speech is represented in terms of the entrained theta oscillations in *B's* speech envelope. Below the line, *A's* prediction of the content of *B's* unfolding utterance is shown as *ĉ*[*sem*, *syn*, *phon*]_*B*_(*t* + 1) and *A's* prediction of *B's* speech timing is shown in terms of theta oscillations in *A's* auditory cortex. The predicted content comes from *A* covertly imitating *B's* utterance at time *t*, deriving *B's* putative production command at time *t+*1 and then feeding this production command into forward models to generate the predictions for time *t+*1. The predicted timing comes from entrainment of *B's* cortical theta oscillations with theta oscillations in *A's* speech envelope.

In conclusion, interlocutors entrain their speech rates based on low-frequency acoustic information. This process appears to be quite separate from the mechanisms of prediction-by-simulation and alignment, which are based on linguistic representations. However, the addressee can combine the results of entrainment (i.e., prediction of timing) with those of linguistic prediction (i.e., prediction of content) to determine the appropriate timing for turn transitions, as we illustrated in the previous section (see Figure 1).

But because the mechanisms of prediction of timing and content are shared with production, we propose that they also aid the addressee's own utterance production. The form of the question (e.g., *What are we going to do after dinner?*) requires a type of answer (verb phrase specifying an activity), which the addressee can prepare by using the same mechanisms that he uses in comprehension. The addressee's onset and rate of articulation follow from the entrainment of speech rate, and specifically the suggestion that such entrainment may also occur in pre-motor cortex (Giraud et al., 2007). This entrainment could therefore be directly applied to the onset and timing of syllable production in relation to the addressee's response, on the assumption that ambient theta oscillations in pre-motor cortex influence the timing of speech articulation.

## Preparing an appropriate response

So far we have concentrated on prediction of content and timing of a partner's current contribution and how this enables the addressee to estimate when the turn will end. But addressees do not merely have to predict content and turn endings; they also have to prepare an appropriate response, or decide not to do so. Recent research has begun to consider the extent to which a responder's planning overlaps with the previous utterance. These studies make use of dual-tasking paradigms (e.g., target tracking or finger tapping) to demonstrate more disruption during production than comprehension (Boiteau et al., 2014; Sjerps and Meyer, 2015). Importantly, the main indication of difficulty during comprehension occurs in about the last half second of the previous utterance, suggesting that planning occurs quite late but is time-locked to turn-ending.

To respond appropriately, the new speaker has to determine the speaker's speech act. For example, a non-rhetorical question mandates a (relevant) answer (or some other valid response such as a query), whereas a rhetorical question does not. Because utterance planning takes time (as we have argued), fluid conversation requires that the addressee should (in general) determine the speech act before the utterance is complete. On occasion, it may not be possible to determine the speech act before the end of the utterance (e.g., because the only relevant information is rising intonation, indicating a question). However, such cases are almost certainly quite rare (Levinson, 2012). For example, the widespread occurrence of *Wh*-words or subject-verb inversion (e.g., *Is the …*) as the beginning of a question provides the addressee with a clear early indication of the speech act. In addition, dialog is full of “pre-sequences” (Schegloff, 1988) that make the upcoming speech act clear well in advance (e.g., *Can I ask you a question?*). Of course, responses are generally congruent with the prior utterance. This is obviously the case for semantics, but is also often true for syntax, as in question-answer pairs (e.g., Levelt and Kelter, 1982) or cross-speaker completions (e.g., Clark and Wilkes-Gibbs, 1986). We propose that comprehenders can make use of this congruency when planning their responses, and use it to share resources between comprehension and preparing production, in accord with Pickering and Garrod (2013a) and in particular the prediction-by-simulation route.

## How addressees take the floor

We have argued that addressees primarily use prediction-by-simulation to predict the content of the speaker's utterance and use prediction-by-simulation in combination with oscillatory entrainment to predict its timing. Prediction of content is enhanced by alignment at many linguistic levels and also facilitates the formulation of an appropriate response. Prediction of timing is used to determine when the speaker will end, and more importantly, when the addressee should start speaking. We now illustrate our account with examples of speaker-addressee turn transition, some of which include difficulties. As the examples show, turn-transition can be entirely straightforward, but very often it leads to minor disruption that can be internally managed (i.e., by the interlocutors themselves); our focus is on spontaneous conversation. Rather more occasionally, it leads to some form of conversational breakdown.

First consider an excerpt from (1) above. Bee describes purchasing an expensive art book and then produces *you know* (highlighted). While hearing this, Ava predicts that Bee is likely to end at this point and that Ava can (or should) take the floor (i.e., this constitutes a potential turn-transition point). The timing of the response is the result of entrainment based on Bee's speech rate. Ava's response *not drop it* reuses part of Bee's previous utterance, as expressed in the words *drop* and *it* and the way they are combined; this repetition occurs because Ava has linguistically aligned with Bee. Interestingly, Bee speaks at the same time as Ava, and produces a semantically equivalent utterance (*hold onto it*). This shows that both Ava's utterance and her timing were appropriate and that her prediction was successful.

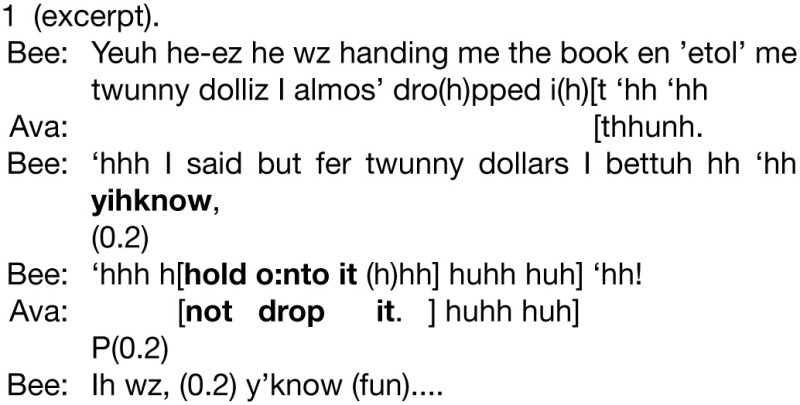



However, Bee's response also creates a problem, because it means that Bee wishes to continue speaking. Ava and Bee's overlap is quite extensive, presumably because they are semantically well aligned (and may therefore find it possible to comprehend and produce three-word overlaps). But they then both produce laughter and stop speaking, before Bee continues. In terms of Pickering and Garrod (2014), after *you know*, Ava predicts that she will say *not drop it* after (say) 300 ms (corresponding to the silence plus laughter). This self-prediction turns out to be correct. Although she may realize what Bee would have said at this point, she presumably does not predict that Bee will also speak at this time, as overlapping speech is strongly disfavored. When Bee does speak, Ava compares her prediction that Bee will not speak with the actual event. This leads to a conflict that could result in her stopping speaking, but in fact she judges that uttering the three words will not be problematic. Similarly, Bee presumably does not predict that Ava will speak at that point, but also judges that continuation would not be problematic. However, the overlap between Ava and Bee can be seen analogous to a speech error (i.e., internal to one speaker) and the laughter, pause, and Bee's eventual continuation can be seen as a form of repair [see points (a) and (g) in discussion of Sacks et al., 1974].

In other cases, the transitions are not quite so successful and require some management. In Example 2 (from Schegloff, 1996, p. 85), Ava begins by describing her unexpected activity^3^.

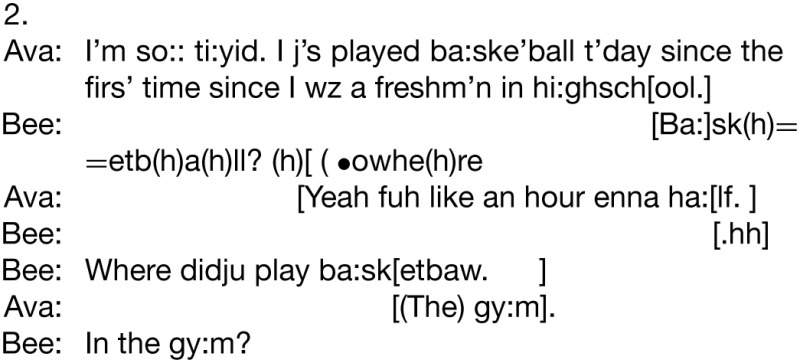



Bee appears to predict that Ava is in the middle of uttering *high school* and about to finish speaking (or at least, reach a turn-transition point). Bee therefore queries *Basketball?*, indicating surprise. Ava appears to interpret Bee's contribution as providing an invitation for Ava to expand, but in fact Bee intends to continue with a more specific question (beginning *where*). So both Ava and Bee predict their own utterance and its timing, but also predict that their partner is not about to speak. When their partner does speak, a clash ensues—with Ava continuing but Bee ceding the floor. However, we propose that Bee retained her question (i.e., her planned utterance) until she was able to predict a turn-transition point (toward the end of *half*) and then produced it. Ava, in turn, predicted that Bee was uttering *basketball* after the first syllable and produced an appropriate response (*the gym*) as Ava was finishing her question. We propose that dealing with these transitions requires speakers to make separate predictions of both content and timing, in accord with our account. (Note that the overlap is twice associated with minor disruption to the first speaker's turn ending, both in *high school* and *basketball*; see Schegloff, 2000).

So far, our examples have been from dyadic interactions. In multi-party conversations, different addressees may be permitted to speak at a turn-transition point. This situation can often lead to short periods of overlapping speech (where there is “competition for the floor”). In (3), Kathy is describing hand-weaving at a dinner party (from Schegloff, 2000, p. 31). After pausing and saying *you know*, both Dave and Rubin speak at the same time. Dave withdraws, and Rubin completes a question to Kathy, who responds to him. We propose that both Dave and Rubin predict that Kathy is about to complete her utterance *you know* and that a response is appropriate. They both predict timing correctly, so that they start immediately after Kathy finishes and therefore at the same time as each other. But neither predicts that the other is about to speak. Hence, there is a large discrepancy between their predictions and what actually happens. (Of course, it is possible that Dave predicts that Rubin would speak but decided to speak anyway, in which case Dave would not encounter such a large discrepancy during monitoring). Dave's approach to this discrepancy is to abandon speech, thus preventing the communicative failure that would likely occur following extended overlap, whereas Rubin's approach is to carry on regardless (perhaps assuming that Dave will give up)^4^.

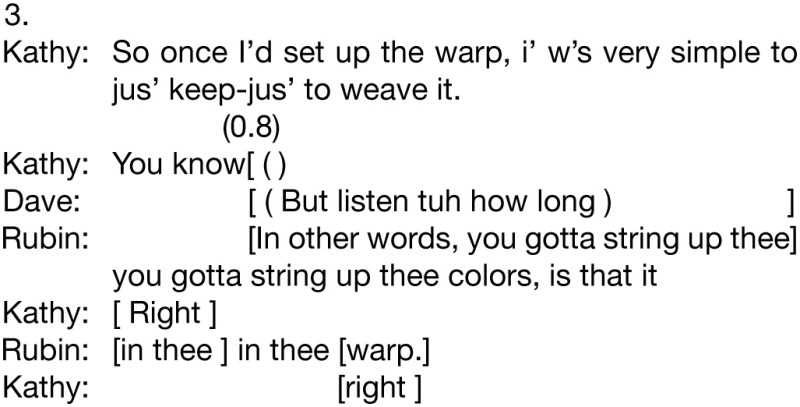



Finally, we note that the addressee can separate the process of prediction from the process of preparing a response. The response can be “ready” before it is executed (just as in Ferreira, 1991; Piai et al., 2011). In (4) (from Schegloff, 2000, p. 25), a family is querying Anne's claim that she used to buy a pair of shoes a month before she was married, and her husband Dick keeps attempting to make a joke about it:^5^

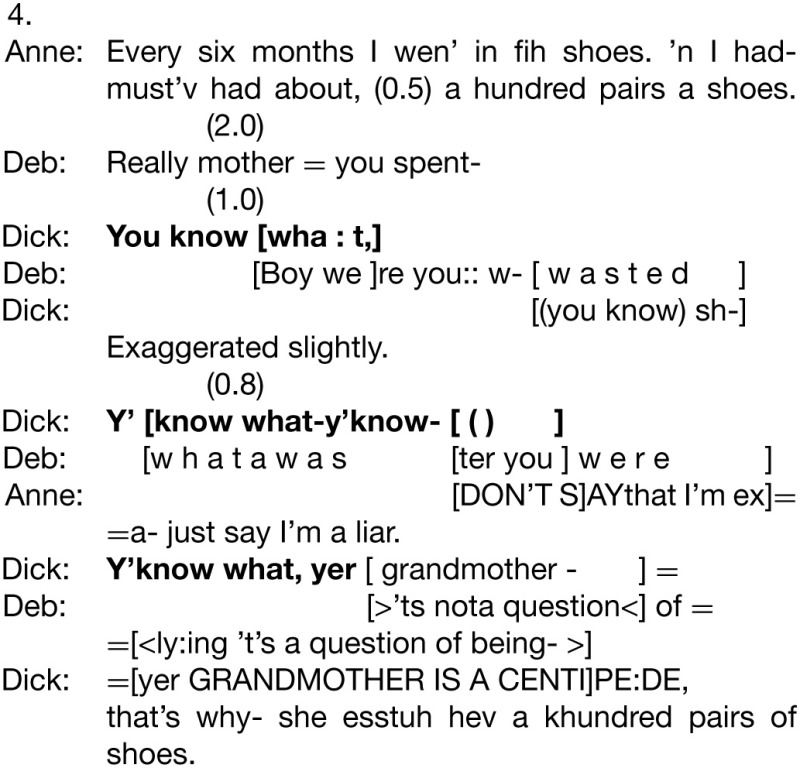



At (24), (29), and (33) (in bold), Dick tries and fails to utter the joke (i.e., Anne is a centipede) that he eventually manages at (36). Dick uses prediction of timing and content to determine a turn-transition point on all four occasions. However, Deb manages to capture the floor three times. On each occasion, Dick has a prepared utterance, which is presumably ready throughout the interchange (from [24] onwards, at least), and hence the preparation of the utterance is separate from the predicted timing. This is a further indication of the distinction between mechanisms for timing and content.

## Implications and discussion

We have shown how content can be combined with timing to predict the end of the interlocutor's turn and determine the appropriate moment to speak. But content and timing can also be used to determine content itself. A good example comes from Dilley and Pitt (2010), who presented listeners with a context spoken at different rates preceding the phrase *leisure or time* and found that they tended to hear it as *leisure time* (i.e., without *or*) if the context was spoken slowly. They then presented listeners with a context preceding the phrase *leisure time* and found that they tended to hear the phrase as *leisure or time* if the context was spoken quickly. Presumably, participants are entrained to the contextual speech rate and then predict that the upcoming phrase will also be produced at that rate. Their interpretation of the phrase is therefore dependent on their predictions. In terms of Figure 1, the predicted timing is used to help determine utterance content.

In this paper, we have focused on the role of prediction during comprehension on turn transition. Specifically, we have argued that comprehenders predict the speaker's content and speech rate, and use these to compute what they are likely to say and how quickly they are likely to say it. We also assume that such prediction helps the comprehender decide when to speak and what to say. However, Pickering and Garrod (2013a) also proposed that prediction during comprehension aids comprehension itself (e.g., facilitating word recognition in noise), aids learning (as comprehenders learn from the discrepancy between the prediction and the actual speech), permits other monitoring (e.g., detecting speaker's errors; Pickering and Garrod, 2014), and assists in the process of alignment (Pickering and Garrod, 2004). Finally, we note that our account is consistent with the effects of timing disruption in dialog. It has been known for 50 years that delaying transmission can seriously disrupt conversation (e.g., Krauss and Bricker, 1967).

A specific set of empirical predictions following from this account concern the separation of timing and content. In a turn-taking paradigm (e.g., question-answering), there should be separate effects of content difficulty (e.g., hard vs. easy questions) and regularity of timing (e.g., varying regularity of speech rate). But in addition, we propose that turn-taking relates to a combination of timing and predicted length in syllables. If a speaker expects a long sentence-final word but gets a short one (e.g., *Is the largest animal in zoo the bear?*, when *elephant* is expected), then the turn interval should be larger than if the expected word was short (*Is the fiercest animal in the zoo the bear*, when *lion* is expected), but this interval should also be affected by speech rate. Experiments such as these should be able to show how predictions of timing and content are separable but ultimately combined in turn-taking.

In conclusion, we have presented a cognitive account to explain the skill with which conversationalists manage turn-transitions in dialog. The account covers addressees' ability to predict when their interlocutor's turn will end, to craft an appropriate response, and to implement the response in a timely fashion. To do this, we propose that they make use of prediction-by-simulation to predict upcoming content and oscillatory entrainment to predict timing. Whereas predicted content depends on forward modeling mechanisms similar to those used in control of speech production, predicted timing results from sensitivity to characteristics of the speech envelope. However, the addressee brings these predictions together in a way that leads to well-coordinated dialog, with very brief turn transitions. In this way, we propose that interlocutors are able to make an apparently difficult aspect of conversation appear remarkably straightforward.

### Conflict of interest statement

The authors declare that the research was conducted in the absence of any commercial or financial relationships that could be construed as a potential conflict of interest.

## Footnotes

^1^Transcription conventions for the original examples in our paper are as follows: (.) indicates brief pause; [indicates overlap; : indicates lengthening;. *hh* indicates long inhalation; (=) at the end of one line and the beginning of the next indicate that the speech is continuous; ( ) indicates that the speech here was unintelligible to the transcriber. Speech between >XXX< is more compressed in pace, speech between <XXX> is more stretched out in pace. Upper case indicates unusually loud speech. In some cases we have added bold font for illustrative purposes.

^2^A more literal rendition of this exchange is:

^3^A more literal rendition of this exchange is:

^4^A more literal rendition of this exchange is:

^5^A more literal rendition of this exchange is:

## References

[B1] Alario F.-X. Hamamé C. M. (2013). Evidence from, and predictions from, forward modeling in language production. Behav. Brain Sci. 36, 348–349. 10.1017/S0140525X1200249X 23789637

[B2] Arnal L. H. Giraud A.-L. (2012). Cortical oscillations and sensory predictions. Trends Cogn. Sci. 16, 390–398. 10.1016/j.tics.2012.05.003 22682813

[B3] Aylett M. Turk A. (2004). The smooth signal redundancy hypothesis: a functional explanation for relationships between redundancy, prosodic prominence, and duration in spontaneous speech. Lang. Speech. 47, 31–56. 10.1177/00238309040470010201 15298329

[B4] Boiteau T. W. Malone P. S. Peters S. A. Almor A. (2014). Interference between conversation and a concurrent visuomotor task. J. Exp. Psychol. Gen. 143, 295–311. 10.1037/a0031858 23421443 PMC3720820

[B5] Chandrasekaran C. Trubanova A. Stillittano S. Caplier A. Ghazanfar A. A. (2009). The natural statistics of audiovisual speech. PLoS Comput. Biol. 5:e1000436. 10.1371/journal.pcbi.1000436 19609344 PMC2700967

[B6] Clark H. H. Wilkes-Gibbs D. (1986). Referring as a collaborative process. Cognition 22, 1–39. 10.1016/0010-0277(86)90010-7 3709088

[B7] Dell G. S. Chang F. (2014). The P-Chain: relating sentence production and its disorders to comprehension and acquisition. Philos. Trans. R. Soc. Lond. B. Biol. Sci. 369:20120394. 10.1098/rstb.2012.0394 24324238 PMC3866424

[B8] DeLong K. A. Urbach T. P. Kutas M. (2005). Probabilistic word pre-activation during language comprehension inferred from electrical brain activity. Nat. Neurosci. 8, 1117–1121. 10.1038/nn1504 16007080

[B9] De Ruiter J. P. Mitterer H. Enfield N. J. (2006). Projecting the end of a speaker's turn: a cognitive cornerstone of conversation. Language 82, 515–535. 10.1353/lan.2006.0130

[B10] Dilley L. C. Pitt M. A. (2010). Altering context speech rate can cause words to appear and disappear. Psychol. Sci. 21, 1664–1167. 10.1177/0956797610384743 20876883

[B11] Ding N. Simon J. Z. (2012). Neural coding of continuous speech in auditory cortex during monaural and dichotic listening. J. Neurophysiol. 107, 78–89. 10.1152/jn.00297.2011 21975452 PMC3570829

[B12] Ding N. Simon J. Z. (2014). Cortical entrainment to continuous speech: functional roles and interpretations. Front. Hum. Neurosci. 8:311. 10.3389/fnhum.2014.00311 24904354 PMC4036061

[B13] Federmeier K. D. (2007). Thinking ahead: the role and roots of prediction in language comprehension. Psychophysiology 44, 491–505. 10.1111/j.1469-8986.2007.00531.x 17521377 PMC2712632

[B14] Ferreira F. (1991). Effects of length and syntactic complexity on initiation times for prepared utterances. J. Mem. Lang. 30, 210–233. 10.1016/0749-596X(91)90004-4

[B15] Ghitza O. (2011). Linking speech perception and neurophysiology: speech decoding guided by cascaded oscillators locked to the input rhythm. Front. Psychol. 2:130. 10.3389/fpsyg.2011.00130 21743809 PMC3127251

[B16] Ghitza O. (2013). The theta-syllable: a unit of speech information defined by cortical function. Front. Psychol. 4:138. 10.3389/fpsyg.2013.00138 23519170 PMC3602725

[B17] Giraud A. L. Kleinschmidt A. Poeppel D. Lund T. E. Frachowiak R. S. J. Laufs H. (2007). Endogenous cortical rhythms determine cerebral specialization for speech perception and production. Neuron 56, 1127–1134. 10.1016/j.neuron.2007.09.038 18093532

[B18] Giraud A. L. Poeppel D. (2012). Cortical oscillations and speech processing: Emerging computational principles and operations. Nat. Neurosci., 15, 511–517. 10.1038/nn.3063 22426255 PMC4461038

[B19] Gross J. Hoogenboom N. Thut G. Schyns P. Panzerri S. Belin P.. (2013). Speech rhythms and multiplexed oscillatory sensory coding in the human brain. PLoS Biol. 11:e1001752. 10.1371/journal.pbio.1001752 24391472 PMC3876971

[B20] Hale J. (2001). A probabilistic early parser as a psycholinguistic model, in Proceedings of the second meeting of the North American Chapter of the Association for Computational Linguistics on Language technologies (Pittsburgh, PA).

[B21] Hickok G. Houde J. Rong F. (2011). Sensorimotor integration of speech processing: computational basis and neural organization. Neuron 69, 407–422. 10.1016/j.neuron.2011.01.019 21315253 PMC3057382

[B22] Indefrey P. Levelt W. J. M. (2004). The spatial and temporal signatures of word production components. Cognition 92, 101–144. 10.1016/j.cognition.2002.06.001 15037128

[B23] Jaeger F. (2010). Redundancy and reduction: Speakers manage syntactic information density. Cogn. Psychol. 61, 23–62. 10.1016/j.cogpsych.2010.02.002 20434141 PMC2896231

[B24] Jungers M. K. Hupp J. M. (2009). Speech priming: evidence for rate persistence in unscripted speech. Lang. Cogn. Process. 24, 611–624. 10.1080/01690960802602241

[B25] Kotz S. A. Schwartze M. (2010). Cortical speech processing unplugged: a timely subcortical-cortical framework. Trends Cogn. Sci. 14, 392–399. 10.1016/j.tics.2010.06.005 20655802

[B26] Krauss R. M. Bricker P. D. (1967). Effects of transmission delay and access delay on the efficiency of verbal communication. J. Acoust. Soc. Am. 41, 286–292. 10.1121/1.1910338

[B27] Levelt W. J. M. Kelter S. (1982). Surface form and memory in question answering. Cogn. Psychol. 14, 78–106. 10.1016/0010-0285(82)90005-6

[B28] Levinson S. C. (2012). Action formation and ascription, in Handbook of Conversational Analysis, eds Sidnell J. Stivers T. (Oxford: Blackwell), 103–130.

[B29] Levy R. (2008). Expectation-based syntactic comprehension. Cognition 106, 1126–1177. 10.1016/j.cognition.2007.05.006 17662975

[B30] MacDonald M. C. (2013). How language production shapes language form and comprehension. Front. Psychol. 4:226. 10.3389/fpsyg.2013.00226 23637689 PMC3636467

[B31] Magyari L. De Ruiter J. P. (2012). Prediction of turn-ends based on anticipation of upcoming words. Front. Psychol. 3:376. 10.3389/fpsyg.2012.00376 23112776 PMC3483054

[B32] Oztop E. Wolpert D. Kawato M. (2005). Mental state inference using visual control parameters. Cogn. Brain Res. 22, 129–151. 10.1016/j.cogbrainres.2004.08.004 15653289

[B33] Piai V. Roelofs A. Schriefers H. (2011). Semantic interference in immediate and delayed naming and reading: attention and task decisions. J. Mem. Lang. 64, 404–423. 10.1016/j.jml.2011.01.004

[B34] Pickering M. J. Garrod S. (2004). Toward a mechanistic psychology of dialogue. Behav. Brain Sci. 27, 169–225. 10.1017/S0140525X04000056 15595235

[B35] Pickering M. J. Garrod S. (2013a). An integrated theory of language production and comprehension. Behav. Brain Sci. 36, 329–392. 10.1017/S0140525X12001495 23789620

[B36] Pickering M. J. Garrod S. (2013b). Forward models and their implications for production, comprehension and dialogue. Behav. Brain Sci. 36, 377–392. 10.1017/S0140525X12003238 24049786

[B37] Pickering M. J. Garrod S. (2014). Self-, other-, and joint monitoring using forward models. Front. Hum. Neurosci. 8:132. 10.3389/fnhum.2014.00132 24723869 PMC3971194

[B38] Sacks H. Schegloff E. A. Jefferson G. (1974). A simplest systematics for the organization of turn-taking for conversation. Language 50, 696–735. 10.1353/lan.1974.0010

[B39] Sahin N. T. Pinker S. Cash S. S. Schomer D. Halgren E. (2009). Sequential processing of lexical, grammatical, and articulatory information within Broca's area. Science 326, 445–449. 10.1126/science.1174481 19833971 PMC4030760

[B40] Schegloff E. A. (1988). Presequences and indirection: applying speech act theory to ordinary conversation. J. Pragmat. 12, 55–62. 10.1016/0378-2166(88)90019-7

[B41] Schegloff E. A. (1996). Turn organization: one intersection of grammar and interaction, in Interaction and Grammar, eds Ochs E. Schegloff E. A. Thompson S. A. (Cambridge: Cambridge University Press), 52–133.

[B42] Schegloff E. A. (2000). Overlapping talk and the organization of turn-taking in conversation. Lang. Soc. 29, 1–63. 10.1017/S0047404500001019

[B43] Sjerps M. J. Meyer A. S. (2015). Variation in dual-task performance reveals late initiation of speech planning in turn-taking. Cognition 136, 304–324. 10.1016/j.cognition.2014.10.008 25522192

[B44] Smith N. J. Levy R. (2013). The effect of word predictability on reading time is logarithmic. Cognition 128, 302–319. 10.1016/j.cognition.2013.02.013 23747651 PMC3709001

[B45] Staub A. Clifton C. Jr. (2006). Syntactic prediction in language comprehension: Evidence from either… or. J. Exp. Psychol. Learn. Mem. Cogn. 32, 425–436. 10.1037/0278-7393.32.2.425 16569157 PMC1479855

[B46] Stivers T. Enfield N. J. Brown P. Englert C. Hayashi M. Heinemann T.. (2009). Universality and cultural specificity in turn-taking in conversation. Proc. Natl. Acad. Sci. U.S.A. 106, 10587–10592. 10.1073/pnas.0903616106 19553212 PMC2705608

[B47] Street R. L. (1984). Speech convergence and speech evaluation in fact-finding interviews. Hum. Commun. Res. 11, 139–169. 10.1111/j.1468-2958.1984.tb00043.x

[B48] Tourville J. A. Guenther F. K. (2011). The DIVA model: A neural theory of speech acquisition and production. Lang. Cogn. Process. 26, 952–981. 10.1080/01690960903498424 23667281 PMC3650855

[B49] Trude A. M. (2013). When to simulate and when to associate? Accounting for inter-talker variability in the speech signal. Behav. Brain Sci. 36, 375–376. 10.1017/S0140525X12002701 23789725

[B50] Van Berkum J. J. A. Brown M. C. Zwitserlood P. Kooijman V. Hagoort P. (2005). Anticipating upcoming words in discourse: evidence from ERPs and reading times. J. Exp. Psychol. Learn. Mem. Cogn. 31, 443–467. 10.1037/0278-7393.31.3.443 15910130

[B51] Wilson M. Wilson T. P. (2005). An oscillator model of the timing of turn-taking. Psychon. Bull. Rev. 12, 957–968. 10.3758/BF03206432 16615316

[B52] Wolpert D. M. (1997). Computational approaches to motor control. Trends Cogn. Sci. 1, 209–216. 10.1016/S1364-6613(97)01070-X 21223909

[B53] Wolpert D. M. Doya K. Kawato M. (2003). A unifying computational framework for motor control and social interaction. Philos. Trans. R. Soc. Lond. B 358, 593–602. 10.1098/rstb.2002.1238 12689384 PMC1693134

[B54] Wolpert D. M. Kawato M. (1998). Multiple paired forward and inverse models for motor control. Neural Netw. 11, 1317–1329. 10.1016/S0893-6080(98)00066-5 12662752

[B55] Zion Golumbic E. M. Ding N. Bickel S. Lakatos P. Schevon C. A.. (2013). Mechanisms underlying selective neuronal tracking of attended speech at a “cocktail party.” Neuron 77, 980–991. 10.1016/j.neuron.2012.12.037 23473326 PMC3891478

